# Atypical Presentation of Morphoea in an Elderly Male: Diagnostic Challenges in the Absence of Autoantibodies and Malignancy

**DOI:** 10.7759/cureus.100764

**Published:** 2026-01-04

**Authors:** Samraiz Nafees, Kristian Galea, Aparna Ravikumar, Adam Bowden, Khaled Zamari, Sophie Hoult, Isabelle Hanson, Abdelrahman Abdalla, Maher Kherbek

**Affiliations:** 1 Internal Medicine, York and Scarborough Teaching Hospitals NHS Foundation Trust, Scarborough, GBR; 2 General Practice, Gloucestershire Hospitals NHS Foundation Trust, Bath, GBR; 3 Pathology, York and Scarborough Teaching Hospitals NHS Foundation Trust, Scarborough, GBR

**Keywords:** connective tissue disorder, histopathology, immunosuppression, localized scleroderma, morphoea, mycophenolate, pruritus, seronegative autoimmune disease

## Abstract

Morphoea, or localized scleroderma, is a rare autoimmune connective tissue disorder distinguished by progressive dermal fibrosis and abnormal collagen deposition. While the condition most commonly affects children and middle-aged women, it can occasionally present in elderly individuals with atypical clinical features. Characteristic presentation includes well-defined plaques of thickened, indurated skin, often accompanied by skin discolouration, dryness, or loss of hair and sweat glands.

This case describes the unusual presentation of morphoea in an 83-year-old male, whose systemic and cutaneous findings closely mimicked paraneoplastic and drug-induced syndromes. Comprehensive serological testing and malignancy screening yielded no evidence of underlying neoplastic or systemic autoimmune disorder. Definitive diagnosis was established through skin biopsy, which revealed histopathological features consistent with morphoea.

This report highlights the importance of maintaining diagnostic vigilance, particularly in the elderly who may present without the expected serological markers or conventional risk factors. The case further highlights the essential role of histopathology in confirming morphoea and differentiating it from other mimicking conditions. Recognition of diverse clinical presentations remains vital for accurate diagnosis and timely management across all age groups.

## Introduction

Morphoea, also referred to as localized scleroderma, is an uncommon autoimmune disorder characterized by immune-mediated inflammation and excessive collagen deposition restricted to the dermis and subcutaneous tissue. Unlike systemic sclerosis, morphoea does not typically involve sclerodactyly, nailfold capillary changes, Raynaud’s phenomenon, or visceral organ involvement [1]. Estimates indicate that the annual incidence is fewer than three people per million, with a significant female predominance [2].

Generally considered benign and self-limiting, morphoea can still result in notable morbidity when lesions are extensive, deepen into underlying tissues, or progress rapidly. The classic presentation involves oval or linear patches of indurated, shiny, hairless skin, most commonly on the trunk and limbs, though deeper involvement and extracutaneous symptoms can occur in more severe subtypes [3]. Diagnosis may be challenging in certain populations, such as the elderly or those with negative serology [4]. This case describes a rare presentation of antibody-negative morphoea in an elderly man, emphasizing the diagnostic uncertainty and complexity encountered in such scenarios.

## Case presentation

**Figure 1:**
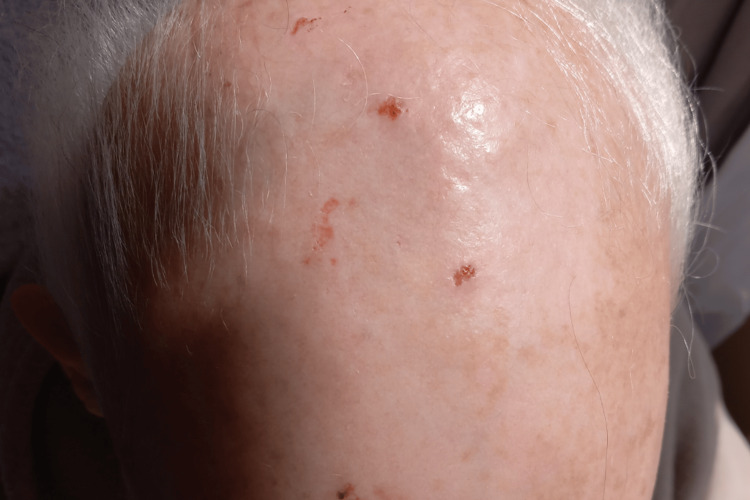
Non-scarring alopecia characterized by diffuse hair thinning over the scalp without permanent follicular damage.

**Figure 2:**
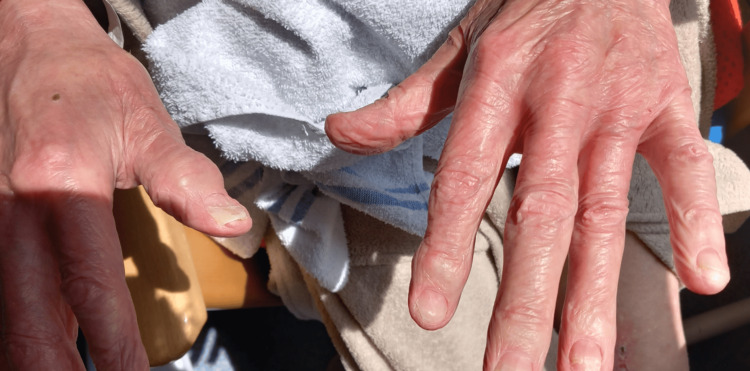
Nail changes: Brittle, dystrophic nails with ridging but preserved nailfolds and normal capillary pattern.

An 83-year-old male presented after a fall with chest pain and vomiting. Over the previous eight months, his symptoms had included bilateral lower limb oedema, intense pruritus, indurated plaques over his abdomen and thighs, progressive skin tightening, and generalized malaise. He had also noted alopecia, loss of nine teeth, and progressive nail dystrophy with brittle, dystrophic nails but normal nailfolds and capillary pattern (Figures 1, 2). Despite the skin changes, there was striking sparing of the hands, feet, and face.

He reported a biphasic Raynaud’s phenomenon for five years without digital ulcers or calcinosis. There was a marked unintentional weight loss of approximately seven stones, accompanied by diminished well-being and chronic itch. His dysphagia had progressed, though a prior barium swallow and oesophagogastroduodenoscopy (OGD) were unremarkable apart from a hiatus hernia and non-erosive gastritis.

His past medical history included type 2 diabetes, gout, chronic heart failure, ischaemic heart disease, Barrett’s oesophagus, and a chronic open left-knee wound post-arthroplasty. He was a non-smoker. Regular medications included bumetanide, paracetamol, gliclazide, and nutritional supplements (Fortisip Compact Protein).

**Figure 3:**
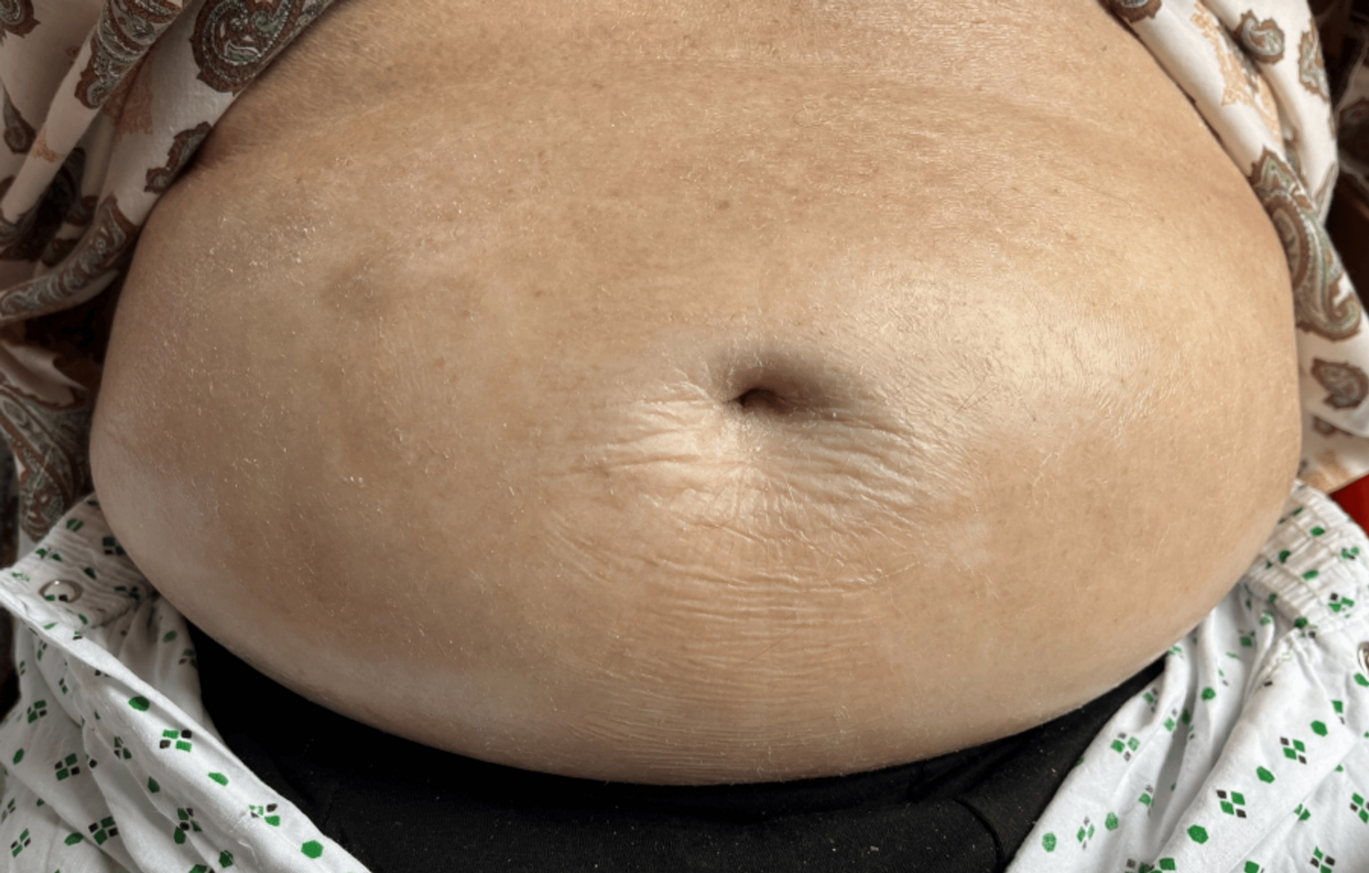
Shiny, thickened, and indurated skin over the lower abdomen with loss of normal skin folds.

Physical examination revealed shiny, thickened, and indurated skin over the lower abdomen, thighs, and upper and lower limbs, with reduced skin folds and subtle extension to the forearms and torso (Figure 3). The hands and feet were warm and well perfused, with no digital ulceration, calcinosis, or sclerodactyly. His facial skin appeared normal. There was mild pitting oedema and generalized pruritus.

**Figure 4:**
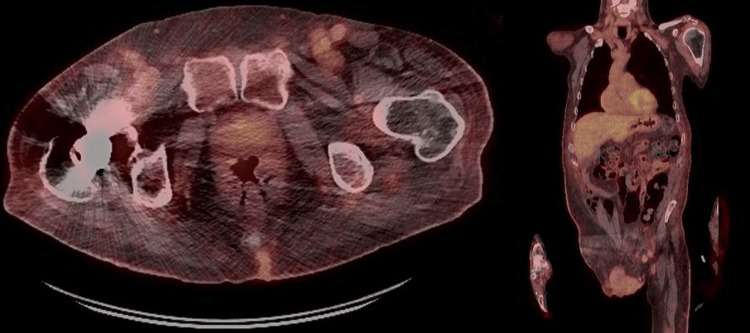
Sagittal and coronal PET images showing mildly increased uptake in the left inguinal and iliac lymph nodes, exceeding background liver activity but remaining below levels typical of high-grade hematologic malignancy, so a low-grade malignant process cannot be excluded.

Investigations revealed normocytic anaemia and transient eosinophilia. Autoimmune serology, including antinuclear antibodies (ANA), extractable nuclear antigen (ENA), antineutrophil cytoplasmic antibodies (ANCA), anti-Scl-70, and an extended scleroderma antibody panel (including anti-centromere, anti-RNA polymerase III, and anti-U3 RNP antibodies), was negative. An echocardiogram indicated pulmonary artery hypertension, with a pulmonary artery pressure of approximately 40 mmHg and right atrial and ventricular dilatation. PET-CT showed a small stable pulmonary nodule, moderate abdominal lymphadenopathy, and gynecomastia (Figure 4). An inguinal node biopsy was performed, which excluded malignancy.

**Figure 5:**
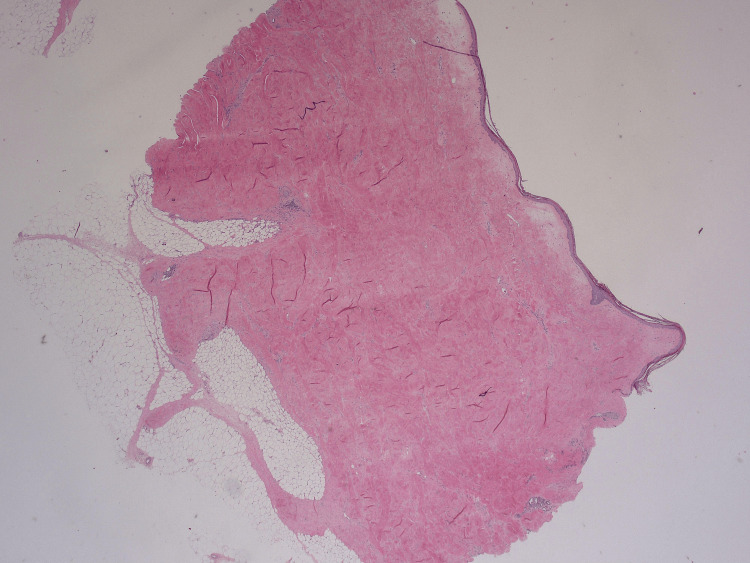
Low-power view of skin biopsy demonstrated a generally collagenized appearance and dermal thickening with adnexal atrophy.

**Figure 6:**
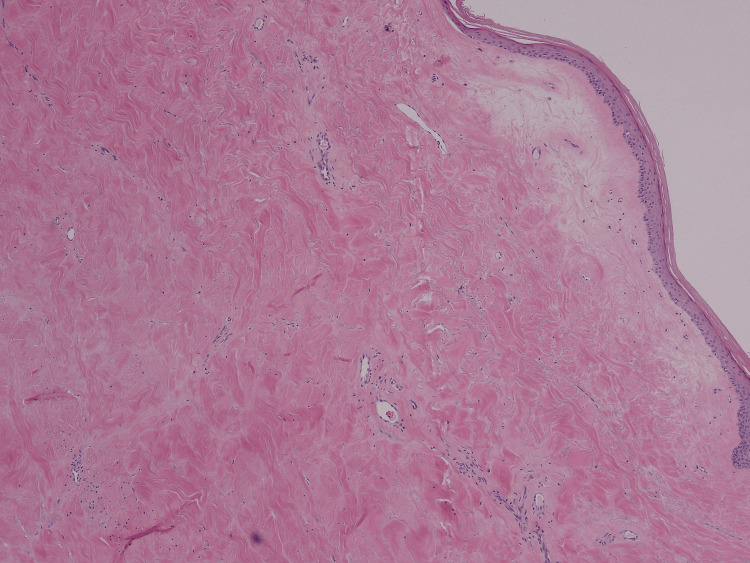
Higher-power view of superficial biopsy, homogeneous, hyalinized dermal collagen.

**Figure 7:**
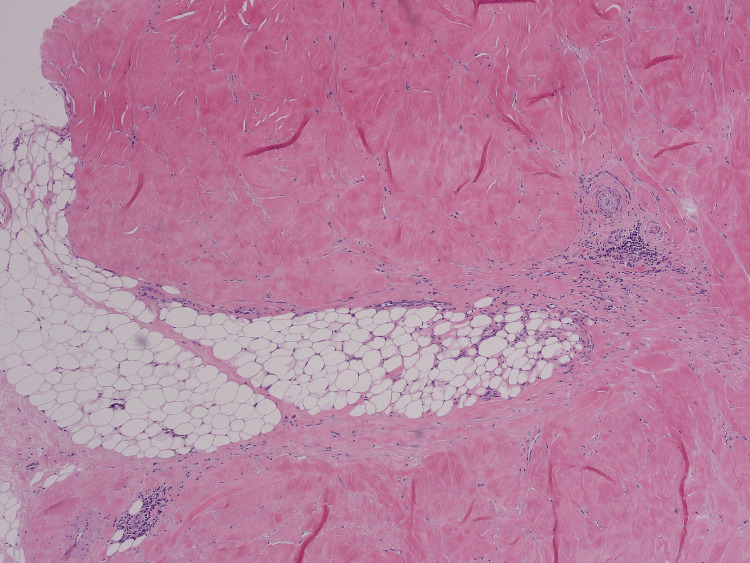
Higher power view of deep dermis, demonstrating collagen extending into subcutis and a mild chronic inflammatory cell infiltrate.

A box-shaped punch biopsy of the abdominal skin demonstrated thickened collagen bundles within the dermis, compression of skin appendages, hyalinization of the epidermis, and a mild chronic inflammatory infiltrate, findings diagnostic of morphoea (Figures 5-7).

Following a multidisciplinary review with dermatology and rheumatology teams, the case was confirmed as antibody-negative morphoea. Differential diagnoses, including DRESS (drug reaction with eosinophilia and systemic symptoms) syndrome, eosinophilic fasciitis, and drug-induced dermatitis, were excluded based on the clinical timeline and histological profile.

Given progressive, symptomatic, and extensive involvement, a plan for systemic immunosuppression with mycophenolate mofetil was made. District nursing support was arranged for blood monitoring and vaccination counselling. He also received emollients, antipruritic management, and wound care advice. Follow-up was scheduled in two weeks with the specialist nursing team and in three months with rheumatology.

## Discussion

This case illustrates an uncommon, antibody-negative presentation of morphoea in an elderly male, with extensive cutaneous and systemic features [5]. While morphoea is typically seen in children and middle-aged women, atypical manifestations, particularly in older male adults, can create significant diagnostic ambiguity [6]. The initial pattern of diffuse induration and generalized symptoms raised suspicion for systemic sclerosis, paraneoplastic syndromes, or drug-related skin conditions. However, negative serology and confirmatory histopathology clarified the diagnosis as localized scleroderma [7]. In contrast, systemic sclerosis typically shows dermal fibrosis extending into subcutaneous tissue with atrophy of adnexal structures, while paraneoplastic and drug-induced skin changes often demonstrate variable inflammatory or interface dermatitis patterns depending on the underlying trigger.

Clinical recognition of morphoea in seronegative individuals hinges on a combination of thorough examination and distinctive histopathological features. In this patient, the deep dermal fibrosis, loss of skin appendages, and surface hyalinization seen on biopsy provided characteristic evidence. Similar histopathological findings may also be observed in conditions such as systemic sclerosis, chronic graft-versus-host disease, sclerodermoid drug reactions, and nephrogenic systemic fibrosis, underscoring the importance of correlating with clinical and serological features [8,9]. Importantly, morphoea rarely affects the hands, feet, or visceral organs, distinguishing it from systemic forms of scleroderma [10].

Extensive morphoea, as in this case, can cause substantial morbidity such as functional limitation, discomfort, and risk of secondary complications, including dental loss, nail dystrophy, or impaired wound healing. While topical therapies suffice for limited disease, systemic treatment is mandatory for progressive cases. Mycophenolate mofetil offers a tolerable alternative to other immunomodulatory agents for frail or elderly patients with comorbidities [11]. However, the prognosis was poor in this patient, and the palliative care team was involved to introduce appropriate supportive and symptom-directed measures.

This case highlights the necessity of considering morphoea as a diagnostic possibility in patients with unexplained cutaneous hardening, regardless of age and serological status [12]. Multidisciplinary management enhances care quality and addresses the often-complex symptom burden in older adults.

## Conclusions

This case highlights the importance of clinical vigilance when assessing antibody-negative morphoea in elderly patients, where overlap with systemic sclerosis or malignancy-related dermatoses can delay diagnosis. Histopathological confirmation remains central to differentiating localized scleroderma from systemic or secondary causes. Individualized, multidisciplinary management, including systemic therapy such as mycophenolate mofetil, can optimize symptom control and quality of life in extensive disease.

## References

[1] Al Badi A, Al-Khamisani M (2024). An unusual presentation of morphea: a case report. Cureus.

[2] Papara C, De Luca DA, Bieber K, Vorobyev A, Ludwig RJ (2023). Morphea: the 2023 update. Front Med (Lausanne).

[3] Knobler R, Moinzadeh P, Hunzelmann N (2017). European Dermatology Forum S1-guideline on the diagnosis and treatment of sclerosing diseases of the skin, part 1: localized scleroderma, systemic sclerosis and overlap syndromes. J Eur Acad Dermatol Venereol.

[4] Sapra A, Dix R, Bhandari P, Mohammed A, Ranjit E (2020). A case of extensive debilitating generalized morphea. Cureus.

[5] Giuggioli D, Colaci M, Cocchiara E, Spinella A, Lumetti F, Ferri C (2018). From localized scleroderma to systemic sclerosis: coexistence or possible evolution. Dermatol Res Pract.

[6] Florez-Pollack S, Kunzler E, Jacobe HT (2018). Morphea: current concepts. Clin Dermatol.

[7] Sahoo D, Devi S, Anupam A, Dash A, Dey A, Mishra B (2025). Paraneoplastic systemic sclerosis: a distinct entity or a mere association - a case report. J Family Med Prim Care.

[8] Foti R, De Pasquale R, Dal Bosco Y (2021). Clinical and histopathological features of scleroderma-like disorders: an update. Medicina (Kaunas).

[9] Walker D, Susa JS, Currimbhoy S, Jacobe H (2017). Histopathological changes in morphea and their clinical correlates: results from the Morphea in Adults and Children Cohort V. J Am Acad Dermatol.

[10] Dańczak-Pazdrowska A, Cieplewicz P, Żaba R, Adamski Z, Polańska A (2021). Controversy around the morphea. Postepy Dermatol Alergol.

[11] Dixit S, Kalkur C, Sattur AP, Bornstein MM, Melton F (2016). Scleroderma and dentistry: two case reports. J Med Case Rep.

[12] Arthur M, Fett NM, Latour E (2020). Evaluation of the effectiveness and tolerability of mycophenolate mofetil and mycophenolic acid for the treatment of morphea. JAMA Dermatol.

